# Bayesian inference for continuous-time hidden Markov models with an unknown number of states

**DOI:** 10.1007/s11222-021-10032-8

**Published:** 2021-08-10

**Authors:** Yu Luo, David A. Stephens

**Affiliations:** 1 Department of Mathematics, Imperial College London, London, UK; 2 Department of Mathematics and Statistics, McGill University, Montreal, Canada

**Keywords:** Bayesian model selection, Continuous-time processes, Hidden Markov models, Markov chain Monte Carlo, Reversible jump algorithms, Model-based clustering

## Abstract

We consider the modeling of data generated by a latent continuous-time Markov jump process with a state space of finite but unknown dimensions. Typically in such models, the number of states has to be pre-specified, and Bayesian inference for a fixed number of states has not been studied until recently. In addition, although approaches to address the problem for discrete-time models have been developed, no method has been successfully implemented for the continuous-time case. We focus on reversible jump Markov chain Monte Carlo which allows the trans-dimensional move among different numbers of states in order to perform Bayesian inference for the unknown number of states. Specifically, we propose an efficient split-combine move which can facilitate the exploration of the parameter space, and demonstrate that it can be implemented effectively at scale. Subsequently, we extend this algorithm to the context of model-based clustering, allowing numbers of states and clusters both determined during the analysis. The model formulation, inference methodology, and associated algorithm are illustrated by simulation studies. Finally, we apply this method to real data from a Canadian healthcare system in Quebec.

**Supplementary Information:**

The online version supplementary material available at 10.1007/s11222-021-10032-8.

## Introduction

Continuous-time Markov processes on a finite state space have been widely used as models for irregularly spaced longitudinal data as they correspond to plausible data generating representations. In almost all cases, the process is observed only at a number of discrete time points, rather than being continuously observed. This problem that arises in a broad collection of practical settings from public health surveillance to molecular dynamics. For example, healthcare systems and electronic health records represent large volumes of data that allow the calculation of longitudinal health trajectories; however, such health records are recorded only when patients interact with the health system. Likelihood-based inference for the infinitesimal generator of a continuous-time Markov jump process has been detailed, for example, in Jacobsen (1982). However, in settings such as those identified above, inference for the infinitesimal generator becomes more difficult. Bladt and Sørensen (2005) investigated likelihood-based inference for discretely observed continuous-time Markov processes, while Tancredi (2019) proposed approximate Bayesian methods to facilitate the computation for such models.

In a related class of problems, the observed data are not directly representative of the Markov process, or similarly the process is observed with measurement error. In those cases, a hidden Markov model (HMM) is more appropriate: this model assumes that an unobserved stochastic process governs the generating model for observations, and assumptions of the Markov property are imposed on the unobserved sequence, with observations usually modeled as independent conditional on the hidden Markov process. There is a broad interest in the application of the continuous-time HMM (CTHMM) in recent years, such as in ecological studies (Mews et al. 2020) and in medical research (Lange et al. 2015; Alaa and Van Der Schaar 2018; Lange et al. 2018), with a predominant focus on frequentist approaches. More recently, Williams et al. (2020) and Luo et al. (2021) implemented a fully Bayesian CTHMM using different missing data likelihood formulations for the underlying Markov chain. Even when these models have been proposed and implemented, the number of states has had to be pre-specified. Determining the number of hidden states is a challenge addressed in earlier work (see for example Celeux and Durand 2008; Pohle et al. 2017). Luo et al. (2021) suggested using the BIC to select the number of states via the expectation–maximization algorithm before performing Bayesian inference with a fixed number of states. Luo et al. (2021) extended Bayesian CTHMMs for finite and Dirichlet mixture model-based clustering procedures to cluster individuals, which allows Markov chain Monte Carlo (MCMC) to change the dimension of the number of clusters, but still relied on the assumption that the number of states has to be pre-specified.

Bayesian model determination approaches have been a longstanding focus of interest in Bayesian inference (see, for example, Carlin and Chib 1995; Green 1995; Godsill 2001). In particular, reversible jump MCMC (Green 1995) has provided a general solution by exploiting trans-dimensional moves that exploit the dynamics of the Metropolis-Hastings (MH) algorithm in a fixed dimension, allowing movement across parameter spaces of different dimensions. Richardson and Green (1997) developed a reversible jump MCMC approach to univariate Normal mixture models, and subsequently Robert et al. (2000) extended this work to discrete-time hidden Markov models with Normal mixtures. In their work, they specifically used two types of reversible jump moves in MCMC to explore the parameter space, i.e., split–combine and birth–death moves. Stephens (2000) introduced an alternative MCMC approach, using a birth–death point process to infer the number of components in the Normal mixture model setting, and Cappé et al. (2003) demonstrated the limit-case equivalence of the reversible jump and continuous-time methodologies for both mixture models and discrete-time HMMs. In this paper, we focus on constructing reversible jump MCMC for CTHMMs which allow the number of hidden states to be inferred via the posterior distribution.

For a better understanding of dynamic changes of individual trajectories, it would be helpful to cluster individuals trajectories and to study the pattern in each group to explore the variation in trajectories. Many of these methods may be classified as model-based clustering procedures, where clustering is achieved by consideration of parametric likelihood- or density-based calculations, with the number of clusters determined by information criteria, such as AIC or BIC (Dasgupta and Raftery 1998; Fraley and Raftery 1998). Similarly, however, in such calculations, the number of clusters has to be fixed, and determining the number of clusters is a challenge addressed by many clustering algorithms. We address this problem subsequently by extending our reversible jump MCMC procedures to allow the number of clusters to be inferred during the analysis.

The rest of the paper is organized as follows. In Sect. 2, we describe the CTHMM-GLM. Section 3 presents fully Bayesian inference via reversible jump MCMC, specifically a split-combine move to update the number of states, and then we update the parameters using fixed dimensional MCMC. Section 4 extends the reversible jump MCMC approach to model-based clustering, allowing numbers of states and clusters to vary simultaneously. Simulation examples to examine the performance of proposed MCMC are presented in Sect. 5. Finally, we present the results for a chronic obstructive pulmonary disease (COPD) cohort in Sect. 6, and discuss these results in Sect. 7.

## A continuous-time hidden Markov model

We presume that the data $\{O_1,\ldots ,O_T\}$ recorded at observation time points $\{\tau _1,\dots ,\tau _T\}$ arise as a consequence of a latent continuous-time Markov chain (CTMC) $\{X_s, s \in \mathbb {R}^+\}$ taking values on the finite integer set $\{1,2,\ldots ,K\}$. Observations are indexed using an integer index (that is, $O_t$), and that the latent process is indexed using a continuous-valued index (that is, $X_{\tau _t}$).

Conditional on the latent process, we assume the observations are drawn from an exponential family model with density $f\left( {O_t} | X_{\tau _t} = k \right)$. If there are time-varying explanatory variables ${\mathbf {Z}}\in \mathbb {R}^D$, a generalized linear model (GLM) with link function *g*(.) and regression coefficients $\beta _{k}$ for state *k*, is adopted. Define matrix ${\mathbf {B}}= (\beta _{d,k})$ for $d=1,\ldots ,D$ and $k =1,\ldots ,K$ as the GLM coefficient matrix. Finally, let $S_t=\left( S_{t,1},\ldots ,S_{t,K}\right) ^\top$ be an indicator random vector with $S_{t,k}=1$ if $X_{\tau _t}=k$ and 0 otherwise.

In the assumed model the data generating mechanism is specified via (i) the latent state model $X_s\left| \varTheta \right. \sim \text {CTMC}\left( \pi ,Q \right)$, where *Q* is the infinitesimal generator and $\pi$ is the initial distribution for the continuous-time Markov process, and (ii) the observation model $O_{\tau _t }\left| X_{\tau _t} = k \right. \sim {\text {GLM}}\left( \beta _{d,k} \right) , d=1,\ldots ,D$. The model is parameterized by $\varTheta =\left\{ Q,\pi ,{\mathbf {B}}\right\}$; recall that the structural constraint on *Q* is that its off-diagonal elements $\{q_{j,k}, j,k=1,\ldots ,K, j \ne k \}$ are positive, and that its rows sum to zero. In this paper, we impose no other constraints, although to do so would be straightforward: for example, we might wish to restrict certain $q_{j,k}$ to obey with linear constraints such as equality to zero. In the model, the observations $\{O_1,\ldots ,O_T\}$ are assumed mutually conditionally independent given $\{X_s\}$; this assumption is common, but can be easily relaxed. With the Markov chain observed discretely at different time points, one could compute the likelihood function for *Q* in Jackson et al. (2003); Williams et al. (2020), however to facilitate the MCMC algorithm, we consider the complete but unobserved trajectory of $\{X_s\}$ as a collection of auxiliary variables in a missing data formulation: the unobserved trajectory comprises a collection of states and transition times that completely describe the latent path over any finite interval. The detailed derivation of the complete data likelihood, ${\mathcal {L}} (\varTheta )$, is given in the Supplement. If there are *N* subjects, let $O_{n,t}$ $\left( t=1,\ldots ,T_n\right)$ be the $t^{\text {th}}$ observation for subject *n* with the associated observation time $\tau _{n,t}$, with the corresponding hidden state $X_{n,\tau _{n,t}}$, and ${\mathbf {O}}_{n}= \{O_{n,1},\ldots ,O_{n,T_n}\}$ and ${\mathbf {X}}_{n} = \{X_{n,\tau _{n,1}},\ldots ,X_{n,\tau _{n,T_n}}\}$ represent the collection of data for subject *n*. ${\mathbf {O}}= \{O_{n,t}\}$ and ${\mathbf {X}}= \{X_{n,\tau _{n,t}}\}$ for $n=1,\ldots ,N, t=1,\ldots ,T_n$ represent the entire data for *N* subjects.

Bayesian inference for this model with the number of states *K* fixed has been fully studied by Luo et al. (2021), where an MCMC scheme based on simulating the complete latent path for each individual is developed; this MCMC scheme relies upon the rejection sampling approach of Hobolth and Stone (2009) to sample the latent paths in an efficient fashion. Bayesian inference using the complete data likelihood formulation is appealing as it produces posterior samples of the full unobserved state sequences and latent continuous-time process, which allows inference to be made for individual-level trajectories across the entire observation window, and which is useful for computing posterior distributions for pathwise aggregate features on individual trajectories.

## Reversible jump MCMC for CTHMMs

First, we add the number of states *K* as an additional parameter and extend the MCMC algorithm to allow for inference to be made via the posterior distribution for *K*. There are several different approaches that can be adopted that we outline below and in the Supplement. First we study split/combine moves for states/pairs of states similar in spirit to the split/merge moves of Richardson and Green (1997); Dellaportas and Papageorgiou (2006). The Supplement also gives detailed descriptions and simulation examples for inference on *K* via a birth-death point process by Stephens (2000).

### Markov chain Monte Carlo methodology

One iteration of the MCMC algorithm that incorporates the required trans-dimensional move proceeds using the following two types of move:

1. A split/combine move that considers splitting a hidden state into two, or combining two hidden states into one.
2. With the number of states *K* fixed, update the model parameters using standard MCMC moves:
  update latent state indicators $\left\{ S_{n,t}\right\}$ for $n=1,\ldots ,N, t=1,\ldots ,T_n$;
  update the parameters associated with the observation process ${\mathbf {B}}$;
  update the initial distribution $\pi$;
  update the infinitesimal generator *Q*.

The Supplement gives detailed procedures of updating the model parameters with a fixed number of states, which was extensively studied in Luo et al. (2021). Specifically, for split and combine moves, we will implement the reversible jump algorithm by Green (1995). Consider a proposal from the current model state $(K,\varTheta )$ to a new state $(K',\varTheta ')$ using the proposal density

$$\begin{aligned} {\mathsf {q}}\left( K',\varTheta ';K,\varTheta \right) = {\mathsf {q}}_1\left( K';K\right) {\mathsf {q}}_2\left( \varTheta _{K'};\varTheta _K\right) \end{aligned}$$

that is, using independent proposals for the two components. The acceptance probability for this form of proposal using the MH procedure is given by

$$\begin{aligned} \begin{aligned}&\alpha \left( K',\varTheta ';K,\varTheta \right) \\&\quad = \min \left( 1, \frac{{\mathsf {q}}\left( K,\varTheta ;K',\varTheta '\right) p\left( K',\varTheta '\left| {\mathbf {o}}\right. \right) }{{\mathsf {q}}\left( K',\varTheta ';K,\varTheta \right) p\left( K,\varTheta \left| {\mathbf {o}}\right. \right) }\right) \\&\quad =\min \left( 1, \frac{{\mathsf {q}}_1\left( K;K'\right) {\mathsf {q}}_2\left( \varTheta _{K};\varTheta _{K'}\right) p\left( K',\varTheta '_{K'}\left| {\mathbf {o}}\right. \right) }{{\mathsf {q}}_1\left( K';K\right) {\mathsf {q}}_2\left( \varTheta _{K'};\varTheta _K\right) p\left( K,\varTheta _K\left| {\mathbf {o}}\right. \right) }\right) \\ \end{aligned} \end{aligned}$$

where $p\left( K,\varTheta _K\left| {\mathbf {o}}\right. \right)$ is the posterior distribution of $(K,\varTheta _K)$ given the observed data ${\mathbf {o}}$, which can, up to proportionality, be decomposed into the marginal (or ‘incomplete data’) likelihood of the data ${\mathcal {L}} ({\mathbf {o}}| \varTheta _K,K )$ times the prior distribution for $(K,\varTheta _K)$;

$$\begin{aligned} p\left( K,\varTheta _K\left| {\mathbf {o}}\right. \right) \propto {\mathcal {L}} ({\mathbf {o}}| \varTheta _K,K ) p_0(\varTheta _K|K) p_0(K) \end{aligned}$$

where $p_0(\cdot )$ represents the prior distribution. Our algorithm relies upon the ability to compute the marginal likelihood efficiently for any $\varTheta _K$; however, this is a standard ‘forward’ calculation for CTHMMs.

### Split and combine moves

To construct efficient split and combine moves under the reversible jump framework, we adopt the idea of centered proposals by Brooks et al. (2003). The proposal is designed to produce similar likelihood contributions for the current and proposed parameters. The combine move is designed to choose a state, *k* at random and select another state $k'$ such that $\sum _{i=1}^{D}\left| \beta _{k,i}-\beta _{j,i}\right|$ is smallest for $j\ne k$. The reverse split move is to randomly select a cluster, *k* to split into two clusters, say *k* and $k'$, and check if the condition, $\sum _{i=1}^{D}\left| \beta _{k,i}-\beta _{k',i}\right| <\sum _{i=1}^{D}\left| \beta _{k,i}-\beta _{j,i}\right|$ for $j\ne k,k'$. If this condition is not met, then the split move is rejected right away.

#### Split move

We consider an update that changes $K \rightarrow K+1$, requiring the generation of a new hidden state. For this move, we set ${\mathsf {q}}_1\left( K;K+1\right) = {\mathsf {q}}_1\left( K+1;K\right)$ for each *K*. Then the acceptance probability reduces to

1 $$\begin{aligned}&\alpha \left( K+1,\varTheta _{K+1};K,\varTheta _K\right) \nonumber \\&\quad =\min \left( 1, \frac{{\mathsf {q}}_2\left( \varTheta _{K};\varTheta _{K+1}\right) p\left( K+1,\varTheta _{K+1}\left| {\mathbf {o}}\right. \right) }{{\mathsf {q}}_2\left( \varTheta _{K+1};\varTheta _K\right) p\left( K,\varTheta _K\left| {\mathbf {o}}\right. \right) }\right) . \end{aligned}$$

We denote the ratio in the final term $r\left( K+1,\varTheta _{K+1};K,\varTheta _K|\right. \left. {\mathbf {o}}\right)$, that is

$$\begin{aligned} r\left( K+1,\varTheta _{K+1};K,\varTheta _K|{\mathbf {o}}\right) = \dfrac{p\left( K+1,\varTheta _{K+1}\left| {\mathbf {o}}\right. \right) }{p\left( K,\varTheta _K\left| {\mathbf {o}}\right. \right) }. \end{aligned}$$

First, we randomly select a state on which to perform the split move. Without loss of generality, we consider the case where state *K* is to be split into new states *K* and $K+1$. We propose the new $(K+1)$-dimensional infinitesimal generator $Q_{K+1}$ using the following updates:

2 $$\begin{aligned} \begin{array}{ccc} q'_{K,j}=q_{K,j} &{} q'_{K+1,j}=q_{K,j} &{} 1\le j<K\\ q'_{i,K}=w_{i}q_{i,K} &{} q'_{i,K+1}=\left( 1-w_{i}\right) q_{i,K} &{} 1\le i <K\\ w_{i} \sim Beta\left( 2,2\right) &{}q'_{K,K+1}, q'_{K+1,K} \sim p_{0Q} \end{array} \end{aligned}$$

with $Q_K= \left\{ q_{i,j}\right\} _{1\le i,j \le K}$ from the original *K*-state model and $Q_{K+1}=\{q'_{i,j}\}_{1\le i,j \le K+1}$; here, $p_{0Q}\left( .\right)$ is the prior distribution for ${q_{i,j}}, \forall 1\le i\ne j\le K$, which is assumed to be $Gamma\left( a,b\right)$. In this way, the new stationary probabilities $s'$ of the CTMC associated with $Q_{K+1}$ satisfying $s'Q_{K+1}=0$ are $s'_j=s_j$ for $1 \le j <K$, $s_K=s'_K+s'_{K+1}$ where *s* is a vector of stationary probabilities associated with $Q_K$ (satisfying $sQ_{K}=0$). The dynamical properties of the CTMC are thus preserved. The observation process parameters associated with new state $K+1$ are generated as

$$\begin{aligned} \beta '_{1,K+1} \sim {\mathcal {N}}\left( \beta _{1,K},c^2\right) , \;\;\; \beta '_{m,K+1}=\beta _{m,K}, \;\; 2\le m \le D \end{aligned}$$

and the remaining elements of ${\mathbf {B}}_{K+1}$ set equal to the elements of ${\mathbf {B}}_{K}$. In addition, we generate a weight $w \sim Beta\left( 2,2\right)$ to split the initial probability for state *K* in $\pi ^K=\left( \pi _1,\ldots ,\pi _K\right) ^\top$ into $\pi '_{K}=w\pi _K$ and $\pi '_{K+1}=(1-w)\pi _{K}$ and the rest remains the same. In the acceptance probability in (1), the ratio of the proposal density can be written as

$$\begin{aligned}&\frac{{\mathsf {q}}_2\left( \varTheta _{K};\varTheta _{K+1}\right) }{{\mathsf {q}}_2\left( \varTheta _{K+1};\varTheta _K\right) }=\frac{{\mathsf {q}}\left( Q_K;Q_{K+1}\right) }{{\mathsf {q}}\left( Q_{K+1};Q_{K}\right) }\times \frac{{\mathsf {q}}\left( {\mathbf {B}}_K;{\mathbf {B}}_{K+1}\right) }{{\mathsf {q}}\left( {\mathbf {B}}_{K+1};{\mathbf {B}}_{K}\right) }\\&\quad \times \frac{{\mathsf {q}}\left( \pi ^K;\pi ^{K+1}\right) }{{\mathsf {q}}\left( \pi ^{K+1};\pi ^{K}\right) }. \end{aligned}$$

Specifically,

$$\begin{aligned} \frac{{\mathsf {q}}\left( Q_K;Q_{K+1}\right) }{{\mathsf {q}}\left( Q_{K+1};Q_{K}\right) }=\frac{\prod \nolimits _{i=1}^{K-1}q_{i,K}}{ p_{0Q} (q'_{K,K+1} )p_{0Q} (q'_{K+1,K} )\prod \nolimits _{i=1}^{K-1}p\left( w_i\right) } \end{aligned}$$

where the numerator comes from the Jacobian of the transformation that creates the proposed $Q_{K+1}$. Then

$$\begin{aligned} \frac{{\mathsf {q}}\left( {\mathbf {B}}_K;{\mathbf {B}}_{K+1}\right) }{{\mathsf {q}}\left( {\mathbf {B}}_{K+1};{\mathbf {B}}_{K}\right) }=\frac{1}{p (\beta '_{1,K} )} \end{aligned}$$

where $p (\beta '_{1,K} )$ is the Normal density with mean $\beta _{1,K}$ and variance $c^2$.

$$\begin{aligned} \frac{{\mathsf {q}}\left( \pi ^K;\pi ^{K+1}\right) }{{\mathsf {q}}\left( \pi ^{K+1};\pi ^{K}\right) }=\frac{\pi _{K}}{p\left( w\right) } \end{aligned}$$

where the numerator comes from the Jacobian of the transformation that generates $\pi ^\prime$. Therefore the MH acceptance probability with the prior distribution as $p_0$ is

3 $$\begin{aligned} \begin{aligned}&\alpha \left( K+1,\varTheta _{K+1};K,\varTheta _K\right) \\&\quad = \min \left( 1, \frac{{\mathsf {q}}\left( Q_K;Q_{K+1}\right) }{{\mathsf {q}}\left( Q_{K+1};Q_{K}\right) } \frac{{\mathsf {q}}\left( {\mathbf {B}}_K;{\mathbf {B}}_{K+1}\right) }{{\mathsf {q}}\left( {\mathbf {B}}_{K+1};{\mathbf {B}}_{K}\right) } \frac{{\mathsf {q}}\left( \pi ^K;\pi ^{K+1}\right) }{{\mathsf {q}}\left( \pi ^{K+1};\pi ^{K}\right) } \right. \\&\quad \left. r\left( K+1,\varTheta _{K+1};K,\varTheta _K|{\mathbf {o}}\right) \right) \\&\quad =\min \left( 1, \frac{d_{K+1}\times \prod \nolimits _{i=1}^{K-1}q_{i,K} \times \pi _{K} }{b_K p_{0Q} (q'_{K,K+1} )p_{0Q} (q'_{K+1,K} )\prod \nolimits _{i=1}^{K-1}p (w_i )p (\beta '_{1,K} ) p\left( w\right) } \right. \\&\quad \left. r\left( K+1,\varTheta _{K+1};K,\varTheta _K|{\mathbf {o}}\right) \right) \\ \end{aligned} \end{aligned}$$

where $b_K$ is the probability of choosing the split move and $d_{K+1}=1- b_K$ is the probability of choosing the combine move.

#### Combine move

For the update from $K+1$ to *K* states, we consider the following move. Without loss of generality, we consider how to combine states *K* and $K+1$ into a single new state *K*. For the current configuration $Q_{K+1}$, we propose the move to $Q_{K}$ as

$$\begin{aligned} \begin{array}{clc} q_{K,j}&{} = \dfrac{s'_K}{s'_K+s'_{K+1}}q'_{K,j}+ \dfrac{s'_{K+1}}{s'_K+s'_{K+1}} q'_{K+1,j} &{} 1\le j<K\\ q_{i,K}&{} = q'_{i,K}+q'_{i,K+1} &{} 1\le i <K \end{array}. \end{aligned}$$

The remaining $q_{ij}$, where $i\ne j$, are obtained by copying $Q_{K+1}$ and discarding $q'_{K,K+1}$ and $q'_{K+1,K}$, with the diagonal terms of $Q_{K}$ calculated by $q_{ii} = -\sum \nolimits _{j\ne i} q_{ij}$ for $1\le i \le K$. It can be verified that the stationary probabilities, $s=\left( s_1,\ldots ,s_K\right) ^\top$ associated with $Q_K$, are $s_j=s'_j$ for $1\le j <K$ and $s_K=s'_K+s'_{K+1}$. For the parameters in observation process, we propose

$$\begin{aligned} \beta _{m,K}=\frac{s'_K}{s'_K+s'_{K+1}}\beta '_{m,K}+\frac{s'_{K+1}}{s'_K+s'_{K+1}}\beta '_{m,K+1} \;\;\;\; 1\le m\le D. \end{aligned}$$

The remaining elements of $\beta _{m,j}$ for $j<K$ are taken to be the same as the current parameter configuration ${\mathbf {B}}_{K+1}$. Finally, we propose the initially distribution $\pi ^{K+1}=\left( \pi '_1,\ldots ,\pi '_K,\pi '_{K+1}\right)$ simply moves to $\pi ^{K}=\left( \pi _1,\ldots ,\pi _K\right)$ where $\pi _K=\pi '_K+\pi '_{K+1}$ and $\pi _j=\pi '_j$ for $j<K$. Therefore, the proposal ratio is computed as follows:

$$\begin{aligned} \frac{{\mathsf {q}}\left( \pi ^{K+1};\pi ^{K}\right) }{{\mathsf {q}}\left( \pi ^K;\pi ^{K+1}\right) }=\frac{p\left( w\right) }{\pi _{K}}. \end{aligned}$$

This is the reverse move corresponding to the split move described above, and essentially $w= \pi '_{K}/\pi _{K}$ and *p*(.) is the density of *Beta*(2, 2). For the infinitesimal generator, the reverse move for $q_{i,K}$ for $1 \le i <K$ is the same with the split move. The reverse move for $q_{K,j}$, $1 \le j <K$, can be viewed as

$$\begin{aligned} q'_{K,j}=\frac{u_1}{u_0}q_{K,j}\;\;\;\;\; q'_{K+1,j}=\frac{1-u_1}{1-u_0}q_{K,j} \end{aligned}$$

where $u_0=s'_K/(s'_K+s'_{K+1})$ and $u_1$ is a weight parameter. If we choose $u_0=u_1$, then $q'_{K,j}=q_{K,j}$ and $q'_{K+1,j}=q_{K,j}$. This reverses what was proposed for the split move. Therefore,

$$\begin{aligned} \frac{{\mathsf {q}}\left( Q_{K+1};Q_{K}\right) }{{\mathsf {q}}\left( Q_K;Q_{K+1}\right) }=\frac{p_{0Q} (q'_{K,K+1} ) p_{0Q} (q'_{K+1,K} )p\left( w_i\right) }{\prod \nolimits _{i=1}^{K-1}q_{i,K}}. \end{aligned}$$

In terms of *B*, since we mimicked the proposal for $q_{K,j}$, therefore the reverse move is $\beta '_{m,K}=\beta _{m,K}$ and $\beta '_{m,K+1}=\beta _{m,K}$ for $1\le m \le D$. Then the proposal ratio ${\mathsf {q}}\left( {\mathbf {B}}_{K+1};{\mathbf {B}}_{K}\right) /{\mathsf {q}}\left( {\mathbf {B}}_K;{\mathbf {B}}_{K+1}\right)$ equals 1, and the MH acceptance probability for the combine move, $\alpha \left( K,\varTheta _K;K+1,\varTheta _{K+1}\right)$, is the minimum of 1 and

4 $$\begin{aligned}&\frac{b_Kp_{0Q} (q'_{K,K+1} )p_{0Q} (q'_{K+1,K} )\prod \nolimits _{i=1}^{K-1}p\left( w_i\right) p\left( w\right) }{d_{K+1} \prod \nolimits _{i=1}^{K-1}q_{i,K} \pi _{K}} \times \nonumber \\&\quad r\left( K,\varTheta _{K};K+1,\varTheta _{K+1}|{\mathbf {o}}\right) . \end{aligned}$$

## Model-based clustering for CTHMMs

So far, we have constructed fully Bayesian inference for a CTHMM via reversible jump MCMC, allowing the number of states to vary during the analysis. We now extend this methodology to cluster trajectories based on a CTHMM with an unknown number of states. Specifically, we will employ model-based clustering procedures to cluster individuals based on the component model parameters that determine the mixture form. The basic formulation of the model envisages that the population is composed of distinct sub-populations each with a distinct stochastic property. For a CTHMM, this corresponds to each group having a potentially different component of parameter $\varTheta = (\pi , Q, {\mathbf {B}})$ and the number of states, *K*. Luo et al. (2021) develop model-based clustering for CTHMMs under finite and infinite mixture models, with a fixed number of states. We incorporate this finite mixture model structure into the proposed reversible jump MCMC, allowing both the number of states and the number of clusters to be inferred during the analysis. There is a crucial distinction between the number of components *M* in the mixture model and the number of clusters $M^*$ in the data which is defined as the number of components used to generate the observed data, or the number of “filled” mixture components. In the algorithm described below, we focus on specifying a prior on the number of components *M*, which implicitly places a prior on $M^*$ (Miller and Harrison 2018); however, in our simulation and real examples, the proposed split move merely generates any empty component. For a comprehensive investigation of *M* and $M^*$ in different trans-dimensional algorithms, see Frühwirth-Schnatter et al. (2020).

Let *M* be the number of components and $C_n$ be the cluster membership indicator for individual *n*. For $n=1,2,\ldots ,N$, it is presumed to be a member of a component labelled $1,2,\ldots ,M$, where $\varpi _m = \mathbb {P}\left( {C_n = m} \right)$ is the prior probability that individual *n* is assigned to component *m*, subject to $\sum \nolimits _{m=1}^M \varpi _M=1$. The following hierarchy leads to model-based clustering procedures for the CTHMM:

$$\begin{aligned}&M \sim p_0\left( M\right) , \text {a mass function on} \left\{ 1,2,3,\ldots \right\} \\&\varpi _1,\ldots ,\varpi _M\left| M\right. \sim \text {Dirichlet}\left( \delta ,\ldots ,\delta \right) \\&\mathbb {P}\left( C_n=m\left| \varpi _1,\ldots ,\varpi _M, M\right. \right) =\varpi _m, m=1,\ldots , M; n=1.\ldots N \\&K_m \sim p_0\left( K\right) , \text {a mass function on} \left\{ 1,2,3,\ldots \right\} \\&{\mathbf {X}}_{n} \left| \varTheta , C_n, K_{C_n} \right. \sim \text {CTMC}\left( \pi ^{(C_n)} ,Q^{(C_n)}\right) \\&{\mathbf {O}}_n \left| {{\mathbf {X}}_{n},\varTheta ,C_n, K_{C_n}} \right. \sim {\text {Exponential Family}}\left( {{B^{(C_n)}}} \right) \end{aligned}$$

with $\delta =1$, making the weight distribution uniform. Then the complete-data likelihood for subject *n* is

$$\begin{aligned}&{\mathcal {L}}\left( C_n,{\mathbf {O}}_n,{\mathbf {X}}_n,\varTheta \right) \\&\quad =\prod \limits _{m=1}^M {\left[ \varpi _m {\mathcal {L}}\left( {\mathbf {O}}_n,{\mathbf {X}}_n \left| C_n=m,\varTheta ^{(m)} ,K_{m} \right. \right) \right] ^{\mathbb {1}\left( C_n=m\right) }} \end{aligned}$$

where $\mathbb {1}\left( C_n=m\right)$ is the indicator function. A subject is assigned to component *m* with a fixed number of states, $K_{m}$, according the posterior probability

5 $$\begin{aligned}&\mathbb {P}\left( C_n=m\left| {{\mathbf {O}}_n,{\mathbf {X}}_n},\varTheta \right. \right) \nonumber \\&\quad =\frac{{{\varpi _m}{\mathcal {L}}\left( {{\mathbf {O}}_n,{\mathbf {X}}_n\left| {C_n = m},\varTheta ^{(m)} \right. } \right) }}{{\sum \nolimits _{l = 1}^M {{\varpi _l}{\mathcal {L}}\left( {{\mathbf {O}}_n,{\mathbf {X}}_n\left| {C_n = l},\varTheta ^{(l)} \right. } \right) } }}. \end{aligned}$$

In reality, the model parameter, $\varTheta$, and the values of the latent states, $X_n$, are not known, and must be inferred from the observed data.

### Reversible-jump MCMC for clustering with an unknown number of states

In Luo et al. (2021), a reversible-jump algorithm based on the marginalized model in (6) was used to update *M*, and we will incorporate this move into our algorithm in Sect. 3 to construct a clustering mechanism which allows the number of clusters and the number of states determined together during the analysis. We first apply the reversible-jump MCMC algorithm in Sect. 3 to update the number of states in each component. We then update the number of components according to a split-combine move, while the combine move only involves components with the same number of states. We summarize one iteration of this clustering mechanism as follows:

1. Update the number of states for each component using the algorithm in Sect. 3.2; If the move is accepted, update the model parameter in the corresponding component.
2. Update the number of components by splitting a component or combining components with the same number of states; If a component with $K_m$ states is chosen in the split move, then the move is to consider splitting the component into two both with $K_m$ states; If two components with the same number of states, $K_m$, are selected in the combine move, then the move is to combine two components into one component with $K_m$ states. Again, we use the idea of centering proposals for the split move, where we fix *Q* and $\pi$ to be the same in the two components, and add some randomness to the intercept in ${\mathbf {B}}$ in the proposed new component. A more detailed explanation is discussed in Sect. 4.2.
3. Given parameters in each component, update the component membership for each individual according to the posterior probability (5).
4. With the number of components *M* fixed, each with fixed states $K_m$ where $m=1,\ldots ,M$, update the model parameters using standard MCMC moves in each component, which the detail is given in the Supplement.

For any empty component from Step 3, we generate model parameters from prior distributions. For the split and combine moves in (b), we carry out them on the marginalized model as in Luo et al. (2021), where component labels and latent processes are marginalized out from the calculation, and use the likelihood

6 $$\begin{aligned} {\mathcal {L}} ({\mathbf {o}}|\varTheta ,M ) = \prod _{n=1}^N \left\{ \sum _{m=1}^M \varpi _m {\mathcal {L}}({\mathbf {o}}_n|\varTheta ^{(m)},K_m ) \right\} . \end{aligned}$$

Similar with updating the number of states, we update *M* by considering a proposal from the current state $(M,\varTheta )$ to a new state $(M',\varTheta ')$ using the proposal density ${\mathsf {q}}\left( M',\varTheta ';M,\varTheta \right) = {\mathsf {q}}_1\left( M';M\right) {\mathsf {q}}_2\left( \varTheta ';\varTheta \right)$, that is, using independent proposals for the two components. The acceptance probability for this proposal is given by

$$\begin{aligned} \begin{aligned}&\alpha \left( M',\varTheta ';M,\varTheta \right) \\&\quad =\min \left( 1, \frac{{\mathsf {q}}_1\left( M;M'\right) {\mathsf {q}}_2\left( \varTheta ;\varTheta '\right) p\left( M',\varTheta '\left| {\mathbf {o}}\right. \right) }{{\mathsf {q}}_1\left( M';M\right) {\mathsf {q}}_2\left( \varTheta ';\varTheta \right) p\left( M,\varTheta \left| {\mathbf {o}}\right. \right) }\right) \\ \end{aligned} \end{aligned}$$

where $p\left( M,\varTheta \left| {\mathbf {o}}\right. \right)$ is the posterior distribution of $(M,\varTheta )$ given the observed data ${\mathbf {o}}$, which can, up to proportionality, be decomposed into the marginal likelihood of the data ${\mathcal {L}} ({\mathbf {o}}| \varTheta ,M )$ times the prior distribution for $(M,\varTheta )$, with prior distribution as $p_0$;

$$\begin{aligned} p\left( M,\varTheta \left| {\mathbf {o}}\right. \right) \propto {\mathcal {L}} ({\mathbf {o}}| \varTheta ,M ) p_0(\varTheta |M) p_0(M). \end{aligned}$$

We will discuss trans-dimensional moves for updating the number of components in more detail below.

### Split/combine move for updating the number of clusters

The combine move is designed to choose a component, *m* say, at random and select another component *i* such that $\left\| {\mathbf {B}}_i-{\mathbf {B}}_m\right\| _2$ is smallest for $i\ne m$. The reverse split move is to randomly select a component, *m* to split into two components, say *m* and $m^*$, and check if the condition, $\left\| {\mathbf {B}}_{m*}-{\mathbf {B}}_m\right\| _2 <\left\| {\mathbf {B}}_j-{\mathbf {B}}_m\right\| _2$ for $j\ne m$. If this condition is not met, then the split move is rejected.

#### Split move

We consider an update that changes the number of component from $M \rightarrow M+1$. Without loss of generality, we aim to split the $M^\text {th}$ component with CTMC parameters $\varTheta _{M}=\{\pi _{M},Q_{M},{\mathbf {B}}_{M}\}$ into two components, requiring the need to generate $K_M$ new hidden states, with corresponding parameters, i.e., $\varTheta {'}=\{\pi {'},Q{'},{\mathbf {B}}{'}\}$ and $\varTheta {''}=\{\pi {''},Q{''},{\mathbf {B}}{''}\}$. To implement the idea of centering proposals, we use a deterministic proposal for *Q* and $\pi$, and let $Q{'}=Q{''}=Q_{M}$ and $\pi ^{'}=\pi ^{''}=\pi _{M}$. For observation parameter ${\mathbf {B}}$, we can use the similar proposal:

$$\begin{aligned}&\beta _{1,k}^{'}=\beta _{M,1,k} \quad \beta _{1,k}^{''}\sim {\mathcal {N}}\left( \beta _{M,1,k},c^2\right) \qquad k=1,\ldots , K_M \\&\beta _{j,k}^{'}= \beta _{j,k}^{''}=\beta _{M,j,k},\; \qquad j=2,\ldots ,D. \end{aligned}$$

For mixture weights $\varpi$, let $w\sim Beta(2,2)$ and define $\varpi {'}=w \varpi _{M}$ and $\varpi {''}=(1-w) \varpi _{M}$. If we define the posterior ratio as

$$\begin{aligned}&r_c\left( M+1,(\varTheta ^{'},\varTheta ^{''},\varpi ',\varpi '');M,(\varTheta _{M},\varpi _M)|{\mathbf {o}}\right) \\&\quad = \dfrac{p\left( M+1,(\varTheta ^{'},\varTheta ^{''},\varpi ',\varpi '')\left| {\mathbf {o}}\right. \right) }{p\left( M,(\varTheta _{M},\varpi _M)\left| {\mathbf {o}}\right. \right) }. \end{aligned}$$

Then, the acceptance probability for this proposal is

7 $$\begin{aligned}&\min \big (1, \frac{{\mathsf {q}}\left( Q_{M};Q^{'},Q^{''}\right) }{{\mathsf {q}}\left( Q^{'},Q^{''};Q_{M}\right) } \frac{{\mathsf {q}}\left( {\mathbf {B}}_{M};{\mathbf {B}}^{'},{\mathbf {B}}^{''}\right) }{{\mathsf {q}}\left( {\mathbf {B}}^{'},{\mathbf {B}}^{''};{\mathbf {B}}_{M}\right) } \frac{{\mathsf {q}}\left( \pi _{M};\pi ^{'},\pi ^{''}\right) }{{\mathsf {q}}\left( \pi ^{'},\pi ^{''};\pi _{M}\right) }\nonumber \\&\quad \frac{{\mathsf {q}}\left( \varpi _{M};\varpi {'},\varpi {''}\right) }{{\mathsf {q}}\left( \varpi {'},\varpi {''};\varpi _{M}\right) }\times \nonumber \\&\quad r_c\left( M+1,(\varTheta ^{'},\varTheta ^{''},\varpi ',\varpi '');M,(\varTheta _{M},\varpi _M)|{\mathbf {o}}\right) \big )\nonumber \\&\quad = \min \left( 1, \dfrac{d_{M+1} \varpi _{M}}{b_M p_{\varpi }(w )p_{\beta }(\beta _{1,k}^{''}) }\times \right. \nonumber \\&\quad \left. r_c\left( M+1,(\varTheta ^{'},\varTheta ^{''},\varpi ',\varpi '');M,(\varTheta _{M},\varpi _M)|{\mathbf {o}}\right) \right) \end{aligned}$$

where $b_M$ is the probability of choosing the split move and $d_{M+1}=1- b_M$ is the probability of choosing the combine move, and $p_{\beta } (\cdot )$ is the Normal density with mean $\beta _{1,K}$ and variance $c^2$ and $p_{\varpi } (\cdot )$ is the *Beta*(2, 2) density.

#### Combine move

For the combine move, we need choose two components with the same number of states and update from $M+1 \rightarrow M$ components. Again, without loss of generality, we consider combine the $(M+1)^\text {th}$ and $M^\text {th}$ components into one component, both components with $K_M=K_{M+1}$ states, with the proposed parameters, $\varTheta {'}=\{\pi {'},Q{'},{\mathbf {B}}{'}\}$. We first find the stationary probabilities, $s_{M}$ and $s_{M+1}$, associated with $Q_{M}$ and $Q_{M+1}$. To combine $Q_{M}$ and $Q_{M+1}$ into $Q^{'}$, the operation is as follows:

$$\begin{aligned}&q_{i,k}^{'}=\frac{s_{M,i}}{s_{M,i}+s_{M+1,i}}\times q_{M,i,k}+\frac{s_{M+1,i}}{s_{M,i}+s_{M+1,i}}\\&\quad \times q_{M+1,i,k}, i\ne k=1,\ldots ,K_M \end{aligned}$$

and $q_{M,k,k}=-\sum _{i \ne k}q_{M,i,k}$ for $k=1,\ldots ,K_M$. For the observation process parameter ${\mathbf {B}}$,

$$\begin{aligned}&\beta _{i,k}^{'}=\frac{s_{M,i}}{s_{M,i}+s_{M+1,i}}\times \beta _{M,i,k}+\frac{s_{M+1,i}}{s_{M,i}+s_{M+1,i}}\\&\quad \times \beta _{M+1,i,k}, i, k=1,\ldots ,K_M. \end{aligned}$$

For the initial distribution $\pi$,

$$\begin{aligned}&\pi _{k}^{'}=\frac{s_{M,i}}{s_{M,i}+s_{M+1,i}}\times \pi _{M,k}+\frac{s_{M+1,i}}{s_{M,i}+s_{M+1,i}}\\&\quad \times \pi _{M+1,k}, k=1,\ldots ,K_M \end{aligned}$$

and rescale the sum to 1. For mixture weights $\varpi$, the move is essentially to add up the probability of the two corresponding components, i.e., $\varpi {'}=\varpi _{M}+\varpi _{M+1}$. The acceptance probability from $M+1$ to *M* components is

8 $$\begin{aligned} \min \left( 1,\frac{b_M p_{\varpi }(w ) }{d_{M+1}\varpi {'}} r_c\left( M,\varTheta {'};M+1,\varTheta _{M},\varTheta _{M+1}|{\mathbf {o}}\right) \right) . \end{aligned}$$

## Simulation

In this section we demonstrate the performance of the proposed reversible jump and birth-death (In the Supplement) MCMC approaches for the CTHMM.

### Identifying the number of states

In the first example, we demonstrate the performance of MCMC to estimate the number of states, and to discover how performance degrades when the problem becomes more challenging. We consider a four-state model with coefficients

$$\begin{aligned} Q=\left( \begin{array}{r@{\quad }r@{\quad }r@{\quad }r} -3.00 &{} 2.00&{} 1.00&{} 0.00\\ 1.00 &{} -1.80 &{}0.75 &{} 0.05\\ 0.15&{} 0.55 &{} -1.05 &{} 0.35 \\ 0.00 &{} 0.25 &{} 0.40&{} -0.65\\ \end{array} \right) \end{aligned}$$

and with time-varying covariates $Z_1\sim {\mathcal {N}}\left( -1,1\right) , Z_2\sim \text {Binomial}\left( 1,0.6\right)$, with

$$\begin{aligned} {\mathbf {B}}=\left( \begin{array}{r@{\quad }r@{\quad }r@{\quad }r} -1.28 &{} -0.55 &{} -1.05 &{} 0.99\\ -0.88 &{} 1.15 &{}1.36 &{} 1.73\\ 0.70 &{}0.68 &{}-1.12 &{}-1.20 \end{array} \right) . \end{aligned}$$

The initial distribution, $\pi$, is set to be $\left( 0.35,0.25,0.2,0.2\right)$. We first construct the continuous time Markov process from the generator *Q* for subject *i*, a continuous-time realization of the latent sequence $\left\{ X_s,0 \le s \le 15\right\}$, and uniformly at random extract *T* observation time points between 0 and 15, where $T \sim Uniform(20,60)$, with the first observation made at time 0. The observations are generated from a Normal or Poisson distribution, with total 1000 subjects. The prior distributions for the elements in *Q* and $\pi$ are specified as independent *Gamma*(1, 2) and $Dirichlet(1,\ldots ,1)$. A non-informative prior is imposed for ${\mathbf {B}}$. We use a zero-truncated *Poisson*(3.5) distribution as the prior for the number of states, and initiate the model with one hidden state.

**Table 1:** Example 5.1: Posterior distribution of the number of hidden states. The true number of states is four

# of hidden states	Normal $\sigma =1$	Normal $\sigma =1.5$	Normal $\sigma =2$	Poisson
1	0.0001	0.0001	0.0001	0.0001
2	0.0005	0.0001	0.0001	0.0002
3	0.0002	0.0002	0.0002	0.0001
4	0.4906	0.3270	0.2534	0.6993
5	0.3845	0.3927	0.3624	0.2506
6	0.1175	0.2237	0.2786	0.0482
7	0.0067	0.0521	0.0928	0.0017
8	0.0000	0.0040	0.0126	0.0000
9	0.0000	0.0003	0.0000	0.0000

The posterior distribution of number of hidden states for different cases are shown in Table 1 with total 20,000 iterations. Trace plots are displayed in the Supplement. In general, the proposed split and combined moves demonstrate desired performance with the trace plots showing that our reversible jump MCMC algorithm has extensively explored the parameter space. In terms of the number of states, the posterior modes for Normal with $\sigma =1$ and Poisson cases are both four with 49.06% and 69.93% respectively, indicating that the proposed MCMC algorithm can identify the number of states where the data are simulated from. However, when we increase $\sigma$ to 1.5 and 2 in Normal case, the posterior modes for the two cases are five with 39.27% and 36.24% respectively, and the percentage of four-state iterations decreased compared to the other two cases. In those cases, the distributions of the number of hidden states are also more diverse, and the MCMC sampler is more likely to explore the higher dimensional parameter space, resulting in fewer iterations of the four-state model.

### Replications and prior sensitivity analysis: identifying the number of states

**Fig. 1:**
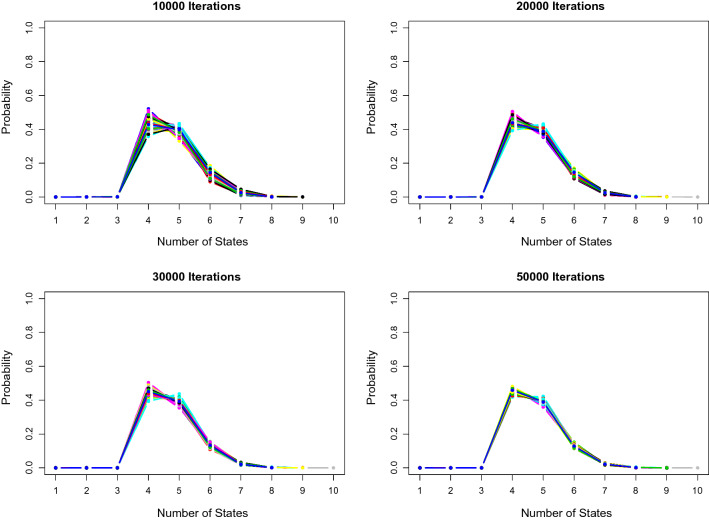
Posterior distribution of the number of states for Normal case $\sigma =1$ with 100 replications for the same dataset

Subsequently, we run 100 replications on the same data set with the same parameter configuration and prior settings as Sect. 5.1 of 500 subjects for Normal case with $\sigma =1$. In each replication, we run 50,000 iterations in total. Figure 1 displays the posterior distribution of the number of states over 100 replications after 10,000, 20,000, 30,000 and 50,000 iterations. In the figure, the proposed RJMCMC algorithm generates consistent results across almost all replications, where the majority of them has the posterior mode four after 50,000 iterations. As the number of iterations increases, the variation of the posterior distribution becomes smaller. After 50,000 iterations, 99 out of 100 replications has the posterior mode four, which demonstrates the stability of the split and combine moves.

**Fig. 2:**
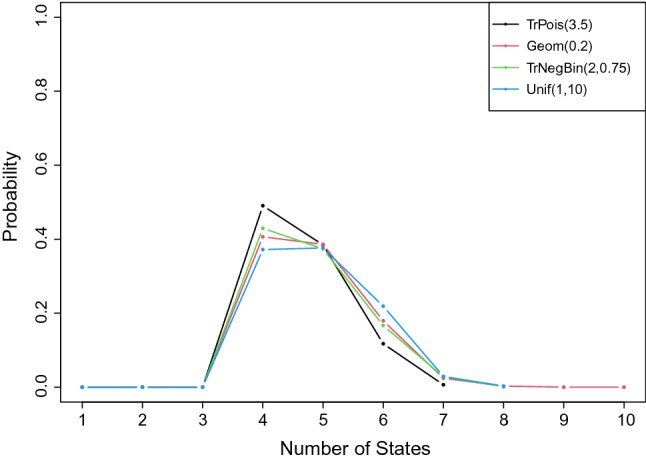
Posterior distribution of the number of states with different prior specifications. TrPois(3.5) represents the zero-truncated Poisson(3.5). Geom(0.2) represents geometric distribution with success probability 0.2. TrNegBin(2,0.75) represents zero-truncated Negative Binomial(2,0.75), and Unif(1,10) represents the discrete uniform distribution

In addition, the prior distribution for *K* can potentially affect the posterior distribution of *K*. Therefore, we run a range of prior distributions for *K* with the same parameter configuration as Sect. 5.1 of 1000 subjects for Normal case with $\sigma =1$, and the results are shown in Fig. 2 based on 20,000 MCMC samples. All cases except Uniform(0,10) have the posterior mode of the number of states at four. The uniform prior yields highly varied posterior distribution, with 37.20%, 37.64% and 21.91% in four, five and six states. While zero-truncated Poisson(3.5) has the smallest variance, the posterior distribution concentrates at four and it puts only little mass on large values of *K*. The zero-truncated Negative Binomial(2,0.75) has the second smallest variance, and we observe the similar result with zero-truncated Poisson. Geometric(0.2) has the largest variance among all the priors, and the posterior distribution is highly varied, ranging from 1 to 10 states. The posterior mass between four and five is small, with probability 0.407 and 0.386 respectively.

### Identifying the number of states: intercept only

In this example, the data are generated with the intercept only in the GLM model. The purpose of this example is to show how the performance differs from previous examples, especially on the values of $\sigma$ in the Normal case. The simulation is configured with three latent states and the associated population generator and the coefficient matrix

$$\begin{aligned} Q=\left( \begin{array}{r@{\quad }r@{\quad }r} -1.0 &{} 0.6&{} 0.4 \\ 0.7 &{} -1.2 &{}0.5 \\ 0.3 &{} 0.6 &{} -0.9\\ \end{array} \right) \end{aligned}$$

with associated coefficient matrices

- Gaussian case: $${\mathbf {B}}=\left( -4,0,5 \right) $$,
- Poisson case: $${\mathbf {B}}=\left( \log (1.5), \log (4), \log (5)\right) $$

The initial distribution $\pi$ for the continuous-time Markov process is set to be $\left( 0.5,0.4,0.1\right)$. As in the first example, we construct the continuous-time Markov process from the generator *Q*, a continuous-time realization of the latent state process $\left\{ X_s,0 \le s \le 15\right\}$, and randomly extract observation time points from the $Uniform\left( 20,60\right)$ between 0 and 15, with the first observation at time 0 in order to estimate the initial probability $\pi$. We generate data for 1000 subjects in each case. The prior distributions for the elements in *Q* and $\pi$ are specified as independent *Gamma*(1, 2) and $Dirichlet(1,\ldots ,1)$. The priors are imposed for the mean of Normal case as ${\mathcal {N}}(0,1)$ and for Poisson case as *Gamma*(10, 10). Again, we use a zero-truncated *Poisson*(3.5) distribution for as the prior for the number of states, and we initiate the model with one hidden state.

**Table 2:** Example 5.3: Posterior distribution of the number of states (Intercept Only). The true number of states is three

# of hidden states	Normal $\sigma =1$	Normal $\sigma =1.5$	Normal $\sigma =2$	Poisson
1	0.0001	0.0001	0.0001	0.0001
2	0.0001	0.0002	0.0002	0.0001
3	0.9823	0.9671	0.9533	0.9765
4	0.0174	0.0328	0.0465	0.0228
5	0.0003	0.0000	0.0000	0.0005

The results are shown in Table 2 with the trace plots of the number of plots in the Supplement. The results presented here are based on 20,000 MCMC samples. As there are fewer parameters in the example, posterior modes for all cases are three, and all cases have over 90% of iterations on the three-state model. Unlike previous cases, the algorithm is less likely to explore higher dimensions compared to models with covariates. We notice that the model constantly visit the four-state model then quickly merged back to three-state model, and this prevents the MCMC sampler to move to a higher dimension. In general, the proposed algorithm shows a good mixing performance in this example.

**Table 3:** Example 5.4: Posterior distribution of the number of cluster with varying states. The true number of clusters is four

# of cluster	Normal $\sigma =1$	Normal $\sigma =1.5$	Normal $\sigma =2$	Poisson
1	0.0001	0.0002	0.0001	0.0001
2	0.0001	0.0003	0.0004	0.0001
3	0.0092	0.0072	0.0029	0.0151
4	0.8706	0.4085	0.3390	0.8713
5	0.1130	0.4354	0.2640	0.1130
6	0.0070	0.1016	0.1930	0.0004
$\ge 7$	0.0000	0.0468	0.2006	0.0000

### Identifying the numbers of clusters and states

In this example, we perform a simulation study to examine the performance of the RJMCMC algorithm for clustering trajectories with varying states. We generate the data from a four-cluster CTHMM, with each cluster having different latent states and specifications

$$\begin{aligned} Q_1= & {} \left( \begin{array}{ccc} -2.5 &{} 2.0&{} 0.5\\ 0.5 &{} -1.5 &{}1.0 \\ 0.1&{} 0.9 &{} -1 \\ \end{array} \right) \; Q_2=\left( \begin{array}{cc} -1.20 &{} 1.20\\ 0.25 &{} -0.25 \\ \end{array} \right) \\ Q_3= & {} \left( \begin{array}{ccc} -0.50 &{} 0.49&{} 0.01\\ 0.25 &{} -0.30 &{}0.05 \\ 0.01&{} 0.10 &{} -0.11 \\ \end{array} \right) \\ Q_4= & {} \left( \begin{array}{cccc} -3.00 &{} 2.00&{} 1.00 &{} 0.00\\ 1.00 &{} -1.80 &{} 0.75 &{} 0.05\\ 0.15 &{} 0.55 &{}-1.05 &{} 0.35\\ 0.00 &{} 0.25 &{} 0.40&{} -0.65\\ \end{array} \right) \end{aligned}$$

with associated coefficient matrices

- Gaussian case: $${\mathbf {B}}_1=\left( -3,0,2\right) $$, $${\mathbf {B}}_2=\left( -3.5,3.5\right) $$, $${\mathbf {B}}_3=\left( -3.8,1,4\right) $$, $${\mathbf {B}}_4=\left( -2,-1.2,0.7,1.8\right) $$.
- Poisson case: $${\mathbf {B}}_1=\left( \log (1.5), \log (4), \log (5)\right) $$, $${\mathbf {B}}_2=\left( \log (2), \log (6)\right) $$, $${\mathbf {B}}_3=\left( \log (1.3), \log (4.2), \log (7.5)\right) $$, $${\mathbf {B}}_4=\left( \log (0.15), \log (0.5),\log (2),\log (6.2)\right) $$.

The initial distributions for three clusters are $\pi _1=\left( 0.5,0.4,\right. \left. 0.1\right)$, $\pi _2=\left( 0.6,0.4\right)$, $\pi _3=\left( 0.45,0.45,0.1\right)$ and $\pi _4=\left( 0.35,0.25,0.2,0.2\right)$. We initiate the model with one cluster with one hidden state. Data are generated by constructing the continuous-time Markov chain from the generator $Q_i$ for cluster $i=1,2,3,4$, a continuous-time realization $\left\{ X_s,0 \le s \le 15\right\}$, and uniformly extract $T-1$ time points between 0 and 15, where $T \sim Uniform\left( 20,60\right)$. Data are generated with 400, 500, 450 and 550 subjects for each cluster respectively. We use the same prior distributions with Sect. 5.1 for the model parameters.

The results presented are based on 10,000 MCMC samples, which is shown in Table 3. Trace plots for the number of clusters for different cases are shown in the Supplement. For the number of clusters, all cases, expect Normal $\sigma =1.5$, have posterior mode four which is the true number of clusters where the data are generated from. For Normal cases, we observe a monotonic decreasing trend for posterior probabilities of four clusters as $\sigma$ increases. The results for the Poisson case are similar to Normal $\sigma =1$. Conditional on four-cluster iterations, trace plots of the number of states display in the Supplement, with missing parts representing non-four-cluster iterations. The posterior modes of the number of states conditional on four-cluster models are consistent with where the data are generated from. For Normal $\sigma =1$ and Poisson cases, trace plots of the number of states are similar to the one-cluster example. For Normal $\sigma =1.5, 2$, we do not observe, in these two cases, mixing as well as previous examples and there are also fewer four-cluster iterations. When $\sigma =1.5$, the posterior modes of the number of states conditional on the four-cluster model are still the consistent with the true data configuration; however, when $\sigma =2$, it is not easy to identify the number of states in each cluster. Compared to previous cases, this is a more difficult problem because of the complexity and the flexibility of the proposed algorithm. For example, when updating the number of clusters, it is less likely to have a successful combine move until two similar clusters have the same number of states. In our example, we set the probability of the combine move for updating the number of clusters as 0.7 to account for issue. Overall, this algorithm performed well in selecting the number of clusters and states in well-separated scenarios.

## Real data analysis: health surveillance of COPD patients

Our real example relates to healthcare surveillance for the chronic condition, COPD, in greater Montreal area, Canada. In 1998, a 25% random sample was drawn from the registry of the Régie de l’assurance maladie du Québec (RAMQ, the Québec provincial health authority) with a residential postal code in the census metropolitan area of Montreal. At the start of every following year, 25% of those who were born in, or moved to, Montreal within the previous year were sampled to maintain a representative cohort. Follow-up ended when people died or changed their residential address to outside of Montreal. This administrative database includes outpatient diagnoses and procedures submitted through RAMQ billing claims, and procedures and diagnoses from inpatient claims.

Using established case-definitions based on diagnostic codes (Lix et al. 2018), COPD patients were enrolled with an incident event occurring after a minimum of two years at risk with no events. Patients were followed from January 1998, starting from the time of their first diagnosis, until December 2014. Physicians only observed these patients during medical visits, which occurred when patients chose to interact with the healthcare system, and at which information, including the number of prescribed medications, is collected. However, as this information was only available for patients with drug insurance, we restrict the cohort to patients over 65 years old with COPD, as prescription data are available for all of these patients. It is widely believed that the progression of COPD can be well-modeled as a progression through a small number of discrete states which approximate severity (GOLD Executive Committee 2017). We are interested in identifying those states and modeling transition between these discrete states, which reflects the performance of the healthcare system over time.

In our analysis, the outcome observations are the number of prescribed medications at the time when patients visited the physician: these are modeled using a Poisson model. In addition, the types of healthcare utilization at each visit were also recorded: hospitalization (HOSP), specialist visit (SPEC), general practitioner visit (GP) and emergency department visit (ER). 4,597 COPD patients are included in this analysis, and these patients are all with drug plans and with at least five years follow-up.

### Identifying the number of states

First, we carry out our analysis to identify the number of states. The analysis is initiated as a one-state model, The prior distributions for the elements in *Q* and $\pi$ are specified as independent *Gamma*(1, 2) and $Dirichlet(1,\ldots ,1)$. We use a zero-truncated *Poisson*(3) distribution for as the prior for the number of states.

#### With covariates

We implement the model including the types of healthcare utilization as covariates in the observation model. A non-informative prior is imposed for ${\mathbf {B}}$. We perform the proposed trans-dimensional MCMC algorithm with 20,000 iterations.

**Table 4:** Application: Posterior distribution of the number of states corresponding to models with and without healthcare utilizations as a covariate in Sects. 6.1.1 and 6.1.2, respectively

# of states	1	2	3	4	5	6	7	8
With Covariates	0.0001	0.0002	0.0002	0.4212	**0.4526**	0.0796	0.0415	0.0045
Without Covariates	0.0001	0.0005	0.3863	**0.4808**	0.1261	0.0064	0.0000	0.0000

The bold value represent the posterior mode of the number of states

**Fig. 3:**
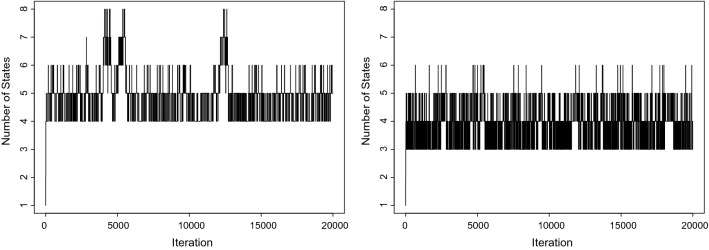
Application: Trace plot for the number of states over 20000 iterations to identify the number of states. The left panel is the observation model with the types of healthcare utilization as covariates, while the right panel is the model without covariates

**Table 5:** Application: Exponential of ${\mathbf {B}}$ coefficients (Parameters in the GLM for each state)

Variables	State 1	State 2	State 3	State 4	State 5
Intercept	2.05	3.53	4.55	6.03	7.52
(95% CI)	(1.80,2.34)	(3.00,4.02)	(3.74,5.32)	(5.35,6.87)	(7.02,8.14)
ED	1.03	1.00	0.99	0.98	0.97
(95% CI)	(1.00,1.06)	(0.98,1.02)	(0.97,1.00)	(0.97,0.99)	(0.95,0.99)
HOSP	1.00	1.00	0.99	0.98	0.96
(95% CI)	(0.95,1.05)	(0.95,1.02)	(0.95,1.01)	(0.97,1.00)	(0.92,0.99)
SPEC	1.02	0.98	0.96	0.97	0.93
(95% CI)	(0.98,1.06)	(0.96,1.01)	(0.94,0.98)	(0.95,0.98)	(0.89,0.96)

Table 4 shows the posterior distribution of the number of states. The trace plot (Fig. 3) confirm that the algorithm has fully explored the parameter space. Although the mode of the posterior distribution of the number of states is five, it also spends over 40% of iterations in the four-state model. Table 5 contains the exponential of the ${\mathbf {B}}$ coefficients condition on the five-state model. On average, from State 1 to 5 the number of drugs taken increases; however, within each state, the numbers of drugs across the different healthcare utilizations are approximately the same. Therefore, it is plausible to consider fitting the intercept-only model without the time-varying covariate, which we will proceed in the next section.

#### Without covariates

We perform the reversible jump trans-dimensional MCMC algorithm for 20,000 iterations without the time-varying covariate, with a *Gamma*(10, 10) distribution placed on the mean number of drugs. Table 4 shows the posterior distribution of the number of states. The trace plot (Fig. 3) confirm that the algorithm has extensively explored the parameter space. Unlike the model with the time-varying covariate, the MCMC algorithm employs most of the time exploring the less complex models, i.e., three-state and four-state model. The posterior mode of the number of hidden states is four. Table 6 contains the expected number of drugs prescribed for patients in each state, with associated 95% credible intervals. As for the model with covariates included, on average, the number of drugs taken increases from State 1 to 4; however, the mean number of drugs prescribed for each state is smaller than the previous five-state model with the time-varying covariate.

**Table 6:** Application: Expected number of drugs for the intercept-only model over the time spent in each state

	State 1	State 2	State 3	State 4
Expected # of Drug Prescribed	3.19	4.00	4.75	5.90
(95% CI)	(2.89,3.31)	(3.29,4.58)	(4.53,5.84)	(5.85,6.05)

### Identifying numbers of clusters and states

Next, we implement the clustering algorithm to group trajectories with distinct stochastic properties. From the previous one-cluster model, we did not observe much distinction across different healthcare utilizations on the number of drugs. Therefore, we decide to cluster patient trajectories using the intercept-only model.

**Fig. 4:**
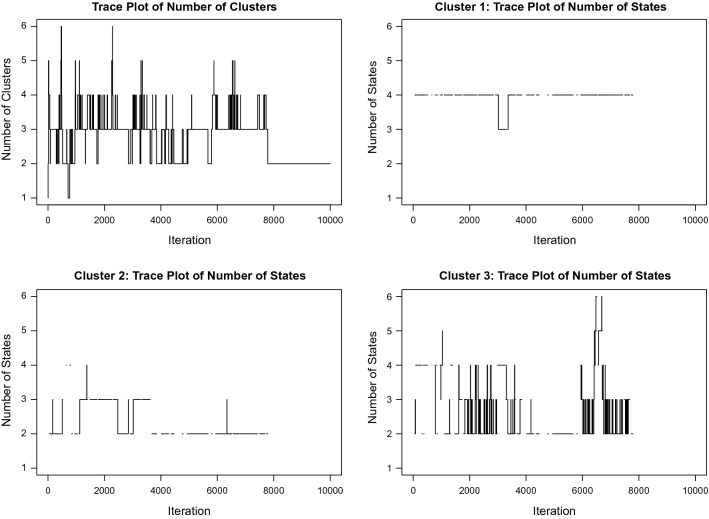
Application: trace plots for the number of clusters and of the number of states on three-cluster iterations

We present results based on 10000 MCMC iterations after initialization from one-cluster model with one hidden state. The mode of the posterior distribution of the number of clusters is three (5358 out of 10000 iterations). Table 7 and Fig. 4 present the posterior distribution and trace plots of the number of clusters and numbers of states conditional on three-cluster iterations. The posterior modes for numbers of states are four, two and two for Cluster 1, 2, 3 respectively. For a summary output, cluster membership is assigned to the subject according to its posterior mode conditional on three-cluster iterations. Table 8 shows the posterior mean of number of drugs for the three-cluster model along with the number of patients in each cluster. Cluster 1 has the greatest number of patients and a posterior mode of four states, which is consistent with results of the one-cluster model in Sect. 6.1. The separation between Cluster 2 and 3 is mainly coming from the parameters in the underlying Markov process, as the $q_{12}$ and $q_{21}$ in Cluster 3 are ten times greater than those in Cluster 2. This suggests that transitions between State 1 and 2 are more frequent in Cluster 3. Also, Cluster 3 on average has the least number of drugs prescribed, indicating that patients in this cluster are possibly on the early stage of COPD.

**Table 7:** Application: Posterior distribution of the number of cluster and numbers of states conditional on three-cluster iterations

	Number of clusters	Number of states		
		Cluster 1	Cluster 2	Cluster 3
1	0.0058	0.0000	0.0000	0.0000
2	0.3678	0.0000	**0.7413**	**0.6480**
3	**0.5358**	0.0418	0.2447	0.1566
4	0.0820	**0.9580**	0.0140	0.1627
5	0.0072	0.0002	0.0000	0.0269
6	0.0014	0.0000	0.0000	0.0058

Bold value represents the posterior modes of the numbers of clusters and states

## Discussion

We have developed a reversible jump MCMC algorithm for the CTHMM-GLM with an unknown number of states and clusters, which is implemented under a fully Bayesian framework. This model can deal with challenges typically encountered in latent multi-state modeling, in particular, irregular visits that vary from individual to individual. Our approach uses a split/combine move to explore the trans-dimensional parameter space, which extended the fixed dimensional MCMC proposed by Luo et al. (2021). Simulation studies demonstrated that the proposed MCMC approach could identify the number of states and the number of clusters from the true data generating mechanism. We were able to implement the developed methods for a real data set from Quebec, Canada, comprising more than four thousand COPD patients tracked over twenty years. Our work demonstrated that with a careful construction of the trans-dimensional proposal, our reversible jump MCMC algorithm can achieve desired performance in term of identifying the number of states and the number of clusters simultaneously.

**Table 8:** Application: Expected number of drugs for the three-cluster Poisson model

		State 1	State 2	State 3	State 4
Cluster 1	# of Drug Prescribed	2.04	3.38	4.79	6.43
$N=4439$	(95% CI)	(1.93,2.26)	(3.04,3.77)	(4.42,5.28)	(6.12,6.79)
Cluster 2	# of Drug Prescribed	3.72	6.62		
$N=135$	(95% CI)	(3.13,4.48)	(4.19,7.74)		
Cluster 3	# of Drug Prescribed	3.15	5.61		
$N=23$	(95% CI)	(2.48,5.47)	(4.09,7.66)		

Focusing on the number of states and the number of clusters, a standard prior specification is adopted exchangeable in form with respect to the state/cluster labels. In the MCMC algorithm, it is possible that the algorithm would potentially suffer from the label-switching problem, which has been addressed by Jasra et al. (2005). In this paper, we primarily considered finite mixture formulations to facilitate the trans-dimensional move between different numbers of states and clusters using the reversible jump MCMC algorithm. Bayesian nonparametric procedures, specifically procedures using Dirichlet process models, have become popular tools to explore the trans-dimensional parameter space, where the models are limiting versions of exchangeable finite mixture models. Dirichlet process models are now widely used in density estimation and clustering, with implementation via MCMC sampling approaches (Neal 2000). In our proposed model, the state space has to be discrete; more generally, there may be health conditions that necessitate the use of a continuous latent process. Bayesian formulations for diffusion or jump processes have been studied in the context of financial data, although such formulations are not common in the analysis of health data, allowing the latent continuous state distribution to have an interpretation as an index or a score. For example, one could use the features included in comorbidity indices to measure multimorbidity in terms of the ability to predict future mortality and health services use. Further studies are needed to address this issue to facilitate the generation of hypotheses about the performance of the healthcare system in managing patients with chronic disease. In addition, our real data analysis focuses on the univariate outcome model; however, the latent process may depend on multiple outcomes. For example, in medical applications, patients over the age of 65 are at high risk of death directly as a result of their disease. Also, patients with multimorbidity have a spectrum of measurements for their physical conditions which joint affects the general health statuses. As discussed in Luo et al. (2021), a specific case is that an additional time-to-event outcome becomes available, e.g., death. We can specifically use a joint modeling framework, which models the joint behavior of a sequence of longitudinal measurements and an associated sequence of event times simultaneously. Another approach, which is more general, is to use separate regressions to model the relevant outcomes but correlated random effects are included among them to account for the intercorrelation. These considerations will be the focus for future research.

## Supplementary Information

Below is the link to the electronic supplementary material.

Supplementary material 1 (zip 34 KB)

Supplementary material 2 (pdf 1683 KB)
