# Supplementary material for: Bayesian inference for continuous-time hidden Markov models with an unknown number of states
Source: Stat Comput. 2021 Aug 10;31(5):57. doi: 10.1007/s11222-021-10032-8 (PMC8550639; doi:10.1007/s11222-021-10032-8)

Noname manuscript No.
(will be inserted by the editor)

Supplementary Material for "Bayesian inference for continuous-time hidden Markov models with an unknown number of states"

Yu Luo · David A. Stephens

Received: date / Accepted: date

1 Likelihood for a Continuous-Time Hidden Markov Model

Figure 1 provides a schematic of the presumed data generating structure for one subject.

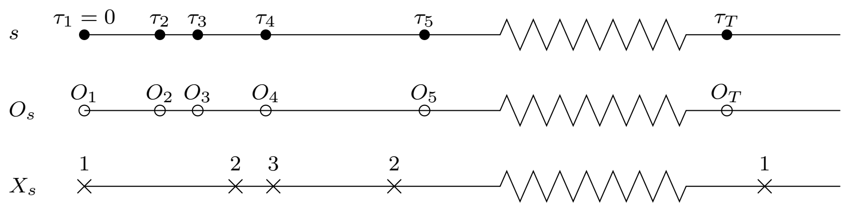

Fig. 1 Schematic of the presumed data generating mechanism for the CTHMM. O_s represents the process underlying the observed data, with observation time points denoted $\tau_t, t = 1, \ldots, T$; X_s represents the hidden Markov process.. Reproduced from Luo et al. (2021).

To construct the complete data likelihood, suppose that $\{X_s\}$ has been observed continuously in the time interval $[0, \tau]$. The likelihood function for Q for individual n is (Bladt and Sørensen, 2005)

$$\mathcal{L}_{n,l,m}(\tau) = \prod_{l=1}^{K} \prod_{m \neq l} {q_{l,m}}^{N_{n,l,m}(\tau)} \exp\left(-q_{l,m} R_{n,l}\left(\tau\right)\right)$$

Y. Luo
Department of Mathematics, Imperial College London, United Kingdom
E-mail: yu.luo@imperial.ac.uk

D. A. Stephens
Department of Mathematics and Statistics, McGill University, Canada
E-mail: david.stephens@mcgill.ca

where $N_{n,l,m}(\tau)$ is the number of transitions from state l to state m in the time interval $[0,\tau]$ and $R_{n,l}(\tau)$ is the total time that the process has spent in state l in $[0,\tau]$ for individual n, and where $q_{l,m}$ are the potentially elements of Q. Note that the quantities $N_{n,l,m}(\tau)$ and $R_{n,l}(\tau)$ are unobserved, but can be computed given a complete realization of the latent process on $[0,\tau]$.

To construct the likelihood for Θ for N independent subjects, we let $O_{n,t}$ $(t = 1,\ldots,T_n)$ be the tth observation for subject n with the associated observation time $\tau_{n,t}$. The complete data likelihood derived from $\{O_n\}$ and $\{X_{n,\tau_n}\}$ can be factorized $\mathcal{L}(\Theta) \equiv \mathcal{L}(\mathbf{O},\mathbf{X}|\Theta) = \mathcal{L}(\mathbf{X}|\Theta)\mathcal{L}(\mathbf{O}|\mathbf{X},\Theta)$ where $\mathbf{O} = \{O_{n,t}\}$ and $\mathbf{X} = \{X_{n,\tau_{n,t}}\}$ for $n = 1,\ldots,N$ and $t = 1,\ldots,T_n$ and

$$\mathcal{L}(\mathbf{O}|\mathbf{X},\Theta) = \prod_{n=1}^{N}\prod_{t=1}^{T_n} f\left(O_{n,t}\,\middle|X_{n,\tau_{n,t}}\right)$$

$$\mathcal{L}(\mathbf{X}|\Theta) = \prod_{n=1}^{N}\pi_{X_{n,0}}\left\{\prod_{t=1}^{T_n-1}\mathcal{L}_{n,l,m}(\Delta_{n,t})\right\}$$

where $\Delta_{n,t} = \tau_{n,t+1} - \tau_{n,t}$. The complete data log-likelihood written in terms of the latent state indicator random vectors $\{S_k\}_{k=1}^{K}$ is

$$\ell(\Theta) = \sum_{n=1}^{N}\sum_{t=1}^{T_n}\sum_{k=1}^{K} S_{n,t,k}\log f\left(O_{n,t}\,\middle|S_{n,t,k}\right) + \sum_{n=1}^{N}\sum_{k=1}^{K} S_{n,1,k}\log\left(\pi_k\right) + \sum_{n=1}^{N}\sum_{t=1}^{T_n-1}\sum_{j=1}^{K}\sum_{k=1}^{K} S_{n,t,j}S_{n,t+1,k}p_{n,t}^{j,k}$$

where

$$p_{n,t}^{j,k} = \sum_{l=1}^{K}\sum_{m\neq l}\left\{N_{n,l,m}^{j,k}\left(\Delta_{n,t}\right)\log\left(q_{l,m}\right) - q_{l,m}R_{n,l}^{j,k}\left(\Delta_{n,t}\right)\right\}.$$

records the probability of transition from state j to state k in the interval $\Delta_{n,t}$, and $N_{n,l,m}^{j,k}(\Delta_{n,t})$ and $R_{n,l}^{j,k}(\Delta_{n,t})$ are the amended versions of $N_{l,m}$ and R_l computed conditional on starting in state j and ending in state k over the interval $\Delta_{n,t}$.

Bayesian inference for this model with the number of states K fixed has been fully studied by Luo et al. (2021), where an MCMC scheme based on simulating the complete latent path for each individual is developed; this MCMC scheme relies upon the rejection sampling approach of Hobolth and Stone (2009) to sample the latent paths in an efficient fashion. Bayesian inference using the complete data likelihood formulation is appealing as it produces posterior samples of the full unobserved state sequences and latent continuous time process, which allows inferences to be made for individual-level trajectories across the entire observation window, and which is useful for computing posterior distributions for pathwise aggregate features on the individual trajectories.

2 Updating Model Parameters for CTHMMs with a Fixed Number of States

For fixed K, we may use a standard a Metropolis-Hastings-within-Gibbs algorithm to generate samples from the posterior distribution for Θ_K. Starting with initial values $\pi^{(0)}$, $\{q_{i,j}\}^{(0)}_{1\leq i\neq j\leq K}$ and $B^{(0)}$, and then given those initial values, the 'forward' and 'backward' values

$$a_{n,t,k} = \mathbb{E}[S_{n,t,k}|\mathbf{o};\Theta_K] = \mathbb{P}(S_{n,t,k}=1|\mathbf{o};\Theta_K) = \sum_{j=1}^{K} b^{\text{old}}_{n,t,k,j}$$

and $b_{n,t,k,j} = \mathbb{P}(S_{n,t,k} = S_{n,t+1,j} = 1|\mathbf{o};\Theta_K)$ are calculated using the forward-backward algorithm (Baum and Eagon, 1967; Baum and Sell, 1968); see Luo et al. (2021) for specific details. At iteration i, the MCMC algorithm simulates the posterior sample based on the full conditional posterior distributions:

- **Update latent state indicators:** For each n and t, generate the random vector $S^{(i)}_{n,t}$ from the multinomial distribution with parameters $a^{(i)}_{n,t} = (a^{(i)}_{n,t,1},\ldots,a^{(i)}_{n,t,K})$.
- **Update B:** Sample coefficient matrix $\mathbf{B}^{(i)}$ and scale parameter $\phi^{(i)}$ given $S^{(i)}_{n,t}$ via the Metropolis-Hastings algorithm as there is no standard distributional forms for the conditional posteriors of $\mathbf{B}$ and ϕ. Proposals are made using a standard Metropolis update from a Normal density for elements of $\mathbf{B}$. Starting values are obtained using an initial GLM fit to the observed data.
- **Update π:** For a conjugate *Dirichlet* $(\alpha_1,\ldots,\alpha_K)$ prior, sample $\pi^{(i)}$ from a Dirichlet distribution with parameters

$$\left(\sum_{n=1}^{N} S^{(i)}_{n,1,1} + \alpha_1, \ldots, \sum_{n=1}^{N} S^{(i)}_{n,1,K} + \alpha_K\right)$$

- **Update Q:** This update is achieved by augmenting the sample space by simulating a path for the latent process. For each n and t,
  - Sample the current state and next state $(X_{n,\tau_{n,t}}, X_{n,\tau_{n,t+1}})$ from a multinomial distribution with the parameter matrix containing the $b_{n,t,k,j}$. Since the likelihood for Q requires continuously observed Markov chain, we first simulate the full path before updating Q.
  - Simulate $N_{n,l,m}\,(\Delta_{n,t})$ and $R_{n,l}\,(\Delta_{n,t})$ from the Markov jump processes step-by-step with infinitesimal generator $Q^{(i-1)}$ through the intervals $[\tau_{n,t},\tau_{n,t+1})$ initiated at $X_{n,\tau_{n,t}}$ and end point $X_{n,\tau_{n,t+1}}$ sampled previously. Simulating sample paths conditional on the endpoints can be achieved efficiently by using modified rejection sampling (that avoids simulating constant sample paths when it is known that at least one state change must take place) as proposed by Hobolth and Stone (2009), from which $\{X_s\}$ is recovered and the jump time points are generated.

– Sample the $\{q_{i,j}\}^{(i)}_{1\leq i\neq j\leq K}$ given the fully recovered Markov process from independent Gamma distributions with shape and rate parameters given as

$$\text{shape} = \sum_{n=1}^{N}\sum_{t=1}^{T_n} N_{n,l,m}\left(\Delta_{n,t}\right) + 1 \qquad \text{rate} = \sum_{n=1}^{N}\sum_{t=1}^{T_n} R_{n,l}\left(\Delta_{n,t}\right).$$

3 Birth-Death MCMC for CTHMMs

In this section, an alternative approach to infer the number of hidden states via a birth-death process is introduced. Instead of constructing a reversible jump approach, Stephens (2000) described a birth-death method, which view each component of the mixture as a point in the parameter space. The birth or death of a state occurs as a marked point process. The detailed description of one iteration of the MCMC algorithm to incorporate the birth-death process is as follows:

1. Given the state of the parameter $\Theta^{(t)}$ at time t, sample the $\Theta^{(t)'}$ by running the birth-death process for a fixed time t_0. Set $K^{(t+1)} = K^{(t)'}$.
2. Fix the number of states K. Update the following parameters given $\Theta^{(t)'}$.
   – Update latent state indicators $S_{n,t}$.
   – Update the parameters associated with the observation process $\mathbf{B}$.
   – Update the initial distribution π.
   – Update the infinitesimal generator Q.

As discussed in Stephens (2000), with a fixed K, Step 2 helps improve the mixing of MCMC.

3.1 Birth-Death Process

The birth or death of a component occurs as a Poisson process. Let the birth rate as λ_b. According to Theorem 3.1 in Stephens (2000), to ensure that a Markov jump process has an invariant probability density which is proportional to the posterior distribution, it is sufficient that

$$\begin{aligned}&p_0\left(K\right)p_0\left(\Theta_K\right)\mathcal{L}\left(K,\Theta_K\right)\mathsf{q}\left(\left(K,\Theta_K\right)\to\left(K+1,\Theta_{K+1}\right)\right)\\&=p_0\left(K+1\right)p_0\left(\Theta_{K+1}\right)\mathcal{L}\left(K+1,\Theta_{K+1}\right)\mathsf{q}\left(\left(K+1,\Theta_{K+1}\right)\to\left(K,\Theta_K\right)\right)\end{aligned}$$

where $q\left(\left(K+1,\Theta_{K+1}\right)\to\left(K,\Theta_K\right)\right)$ is the proposal density from $(K+1,\Theta_{K+1})\to(K,\Theta_K)$. Hence, the corresponding death rate for State K, given $K+1$ states, should be

$$\delta_j = \lambda_b \frac{p_{0Q}\left(q'_{K,K+1}\right)p_{0Q}\left(q'_{K+1,K}\right)\prod_{i=1}^{K-1} p\left(w_i\right)p\left(w\right)}{\prod_{i=1}^{K-1} q_{i,K}\times\pi_K} \times r\left(K,\Theta_K;K+1,\Theta_{K+1}|\mathbf{o}\right)$$

Table 1 BDMCMC: Posterior distribution of the number of states (Intercept Only)

t_0	# of hidden states	Normal $\sigma = 1$	Normal $\sigma = 1.5$	Normal $\sigma = 2$	Poisson
10^{-15}	1	0.0001	0.0001	0.0001	0.0001
	2	0.0001	0.0001	0.0001	0.0002
	3	0.4508	0.2096	0.3313	0.2967
	4	0.4932	0.4529	0.4758	0.4704
	5	0.0506	0.2900	0.1762	0.2023
	6	0.0054	0.0470	0.0166	0.0298
	7	0.0000	0.0004	0.0001	0.0008
10^{-20}	1	0.0001	0.0001	0.0001	0.0001
	2	0.0001	0.0001	0.0001	0.0001
	3	0.4529	0.3899	0.3334	0.4083
	4	0.4976	0.4881	0.4798	0.4904
	5	0.0481	0.1122	0.1677	0.0920
	6	0.0014	0.0096	0.0191	0.0091
	7	0.0000	0.0001	0.0000	0.0000

The total death rate is $\delta_d = \sum_{j=1}^{K+1} \delta_j$. Once a birth or death happens, the time to next birth or death follows an exponential distribution with rate $\delta_d + \lambda_b$, with respective probabilities

$$\mathbb{P}\,(\text{birth move}) = \frac{\lambda_b}{\delta_d + \lambda_b} \qquad \mathbb{P}\,(\text{death move}) = \frac{\delta_d}{\delta_d + \lambda_b}$$

– If the birth move is simulated, sample the new component parameters according to the split move in reversible jump MCMC.
– If the death move is simulated, select a hidden state to ‘die’ with probability δ_j/δ_d where $j = 1, \dots, K+1$.
– Simulate the time to the next jump from an exponential distribution with rate $\delta_d + \lambda_b$.

4 Simulation: Birth-Death MCMC

We used the exactly the same data generating mechanism in Section 5.3 in the main paper. The prior distributions are also the same with that example. We initiate the model with one hidden state. We update the number of hidden states using the birth-death process with a fixed time t_0, with a birth rate $\lambda_b = 1$.

The trace plots of the number of states for all the cases are shown in Figure 2 with the corresponding posterior distribution Table 1. As shown in the trace plots, the number of states change more frequently than reversible-jump MCMC. With a smaller t_0, the posterior distribution is more concentrated on three and four. However, in all cases, the posterior modes are four instead of three (the true number of states which the data were generating from); however, the posterior probabilities between three and four state models are close.

5 Simulation: Trace Plots

References

Baum, L. and J. Eagon (1967). An inequality with applications to statistical estimation for probabilistic functions of Markov processes and to a model for ecology. *Bulletin of the American Mathematical Society 73*, 360–363.

Baum, L. and G. Sell (1968). Growth transformations for functions on manifolds. *Pacific Journal of Mathematics 27*, 211–227.

Bladt, M. and M. Sørensen (2005). Statistical inference for discretely observed Markov jump processes. *Journal of the Royal Statistical Society: Series B (Statistical Methodology) 67*(3), 395–410.

Hobolth, A. and E. A. Stone (2009). Simulation from endpoint-conditioned, continuous-time Markov chains on a finite state space, with applications to molecular evolution. *The Annals of Applied Statistics 3*(3), 1204–1231.

Luo, Y., D. A. Stephens, and D. L. Buckeridge (2021). Bayesian clustering for continuous-time hidden Markov models. *arXiv preprint arXiv:1906.10252*.

Luo, Y., D. A. Stephens, A. Verma, and D. L. Buckeridge (2021). Bayesian latent multi-state modeling for non-equidistant longitudinal electronic health records. *Biometrics 77*(1), 78–90.

Stephens, M. (2000). Bayesian analysis of mixture models with an unknown number of components—an alternative to reversible jump methods. *The Annals of Statistics 28*(1), 40–74.

Fig. 2 Trace Plots for the Number of States of 20000 Iterations using BDMCMC with $t_0 = 10^{-15}$ (left panel) and $t_0 = 10^{-20}$ (right panel).

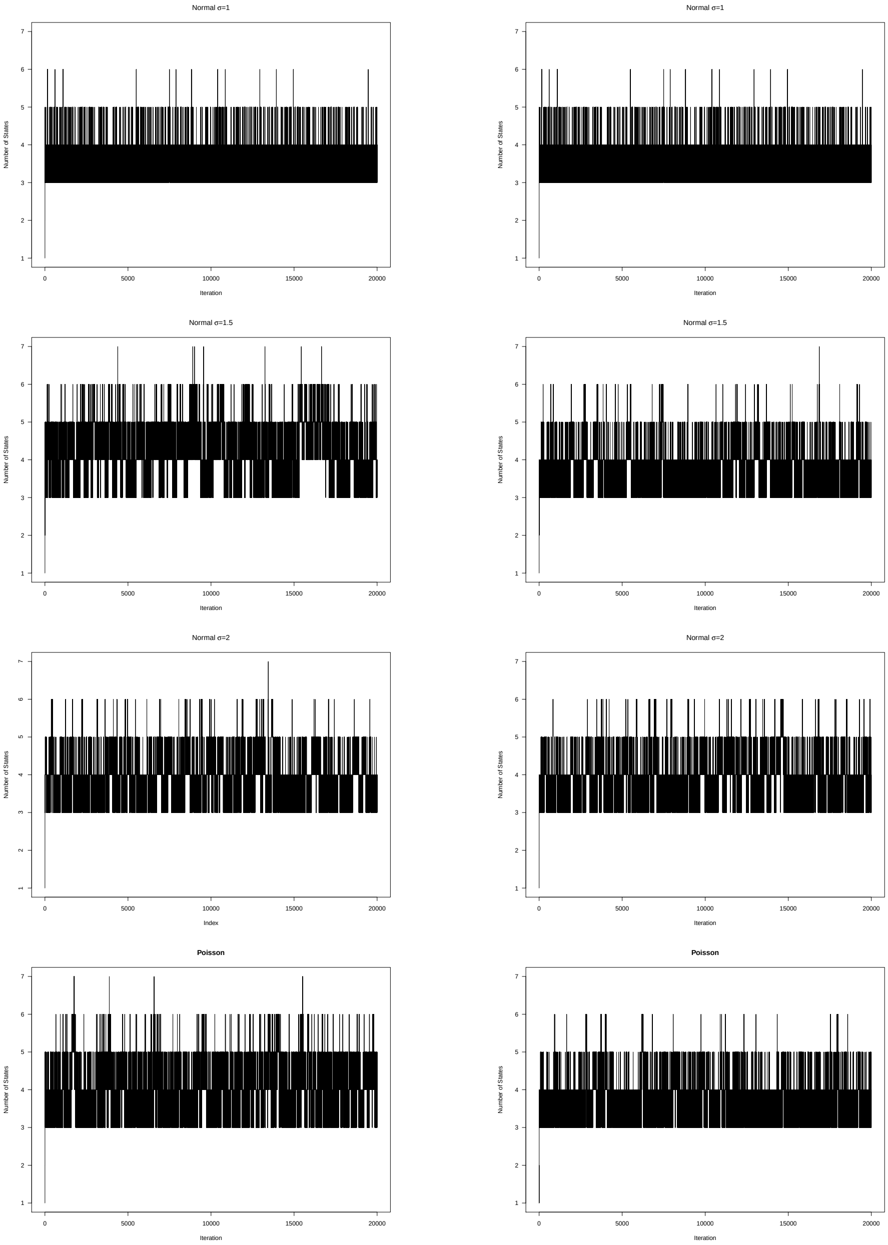

Fig. 3 Example 1: Trace Plots for the Number of States of 20000 Iterations

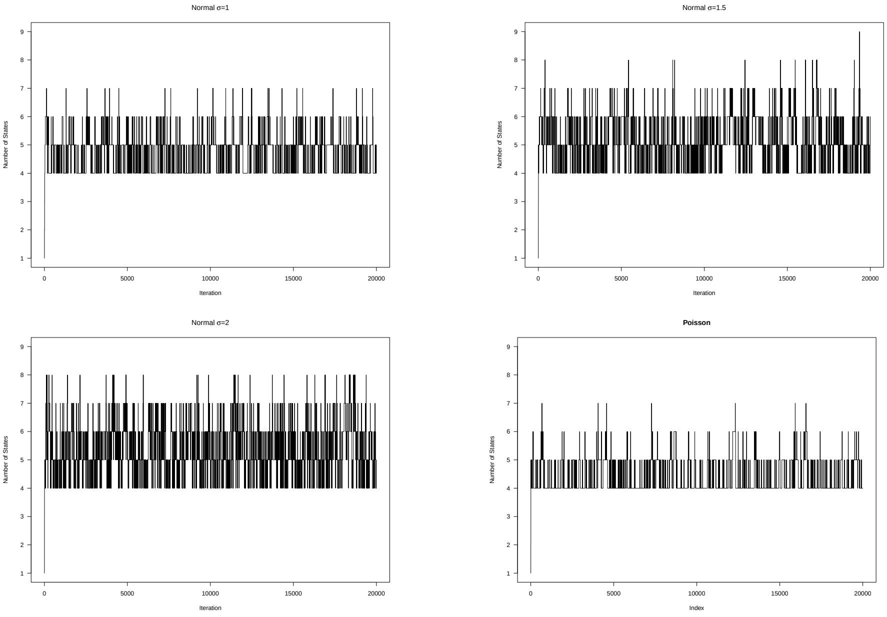

Fig. 4 Example 3: Trace Plots for the Number of States of 20000 Iterations (Intercept only)

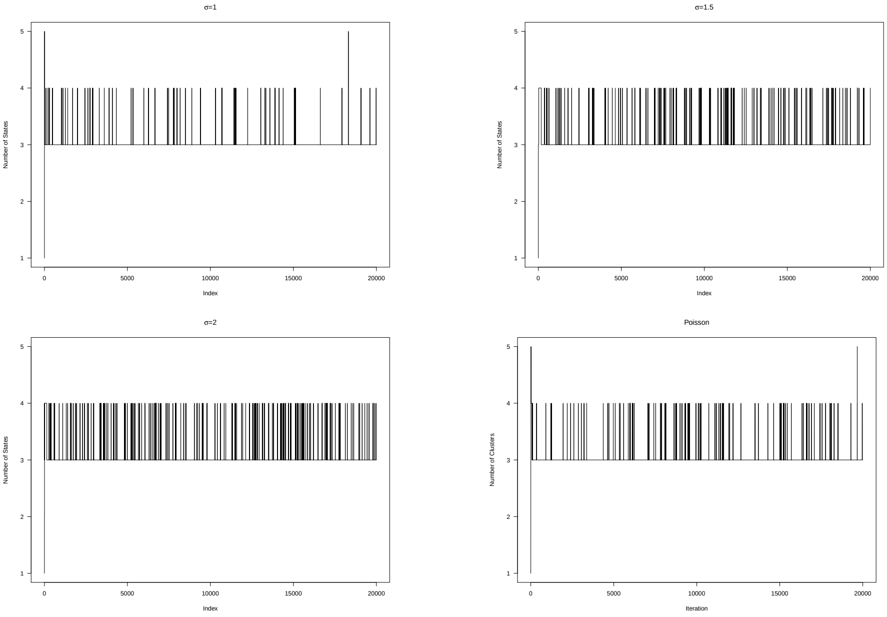

Fig. 5 Example 4: Trace Plots for the Number of Clusters of 10000 Iterations.

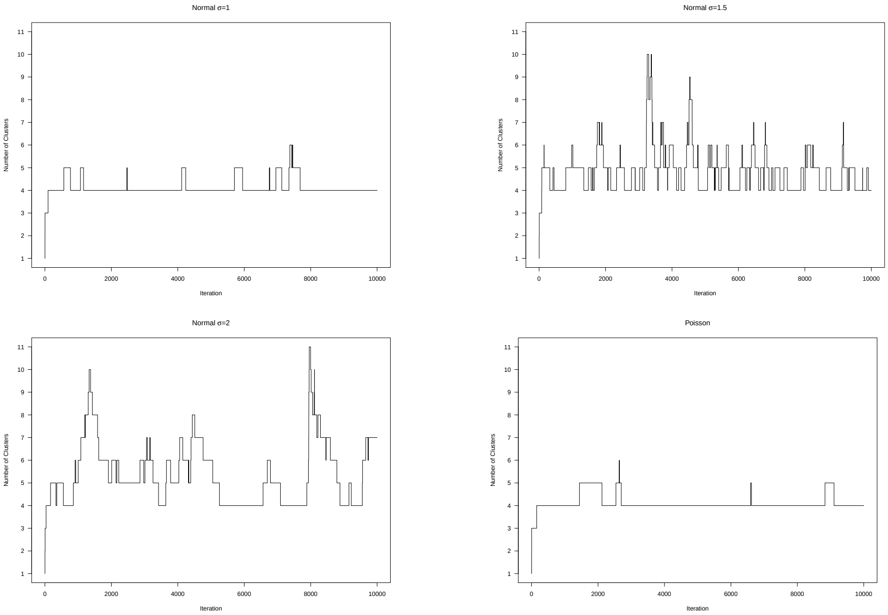

Fig. 6 Example 4: Trace plots of the number of states for Normal case $\sigma = 1$ on four-cluster iterations.

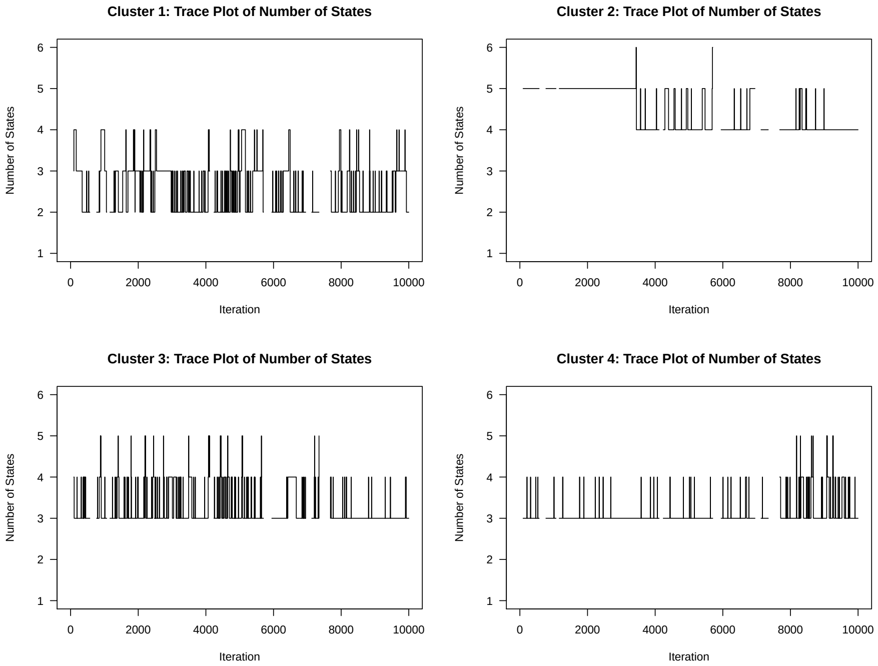

Fig. 7 Example 4: Trace plots of the number of states for Normal case $\sigma = 1.5$ on four-cluster iterations.

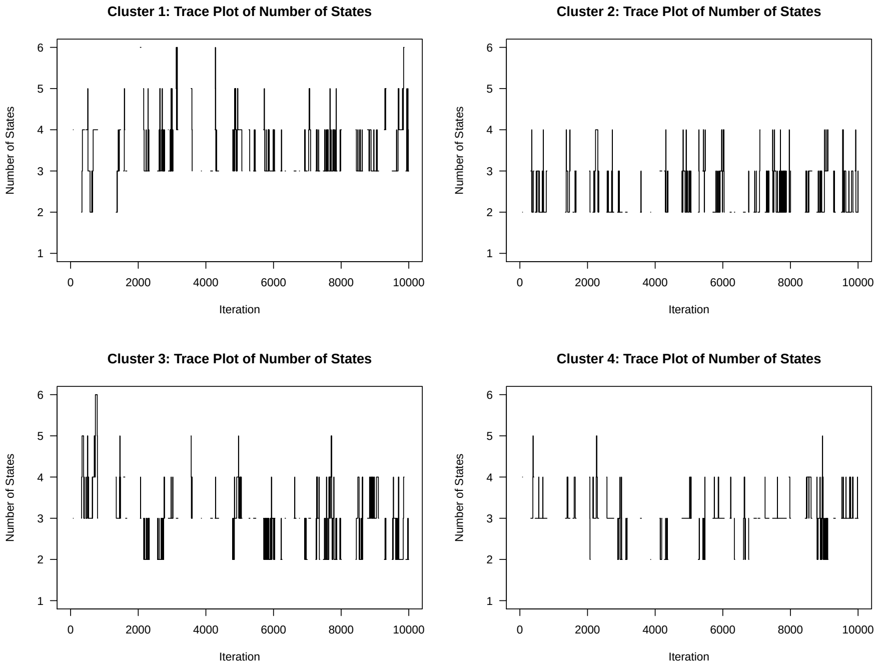

Fig. 8 Example 4: Trace plots of the number of states for Normal case $\sigma = 2$ on four-cluster iterations.

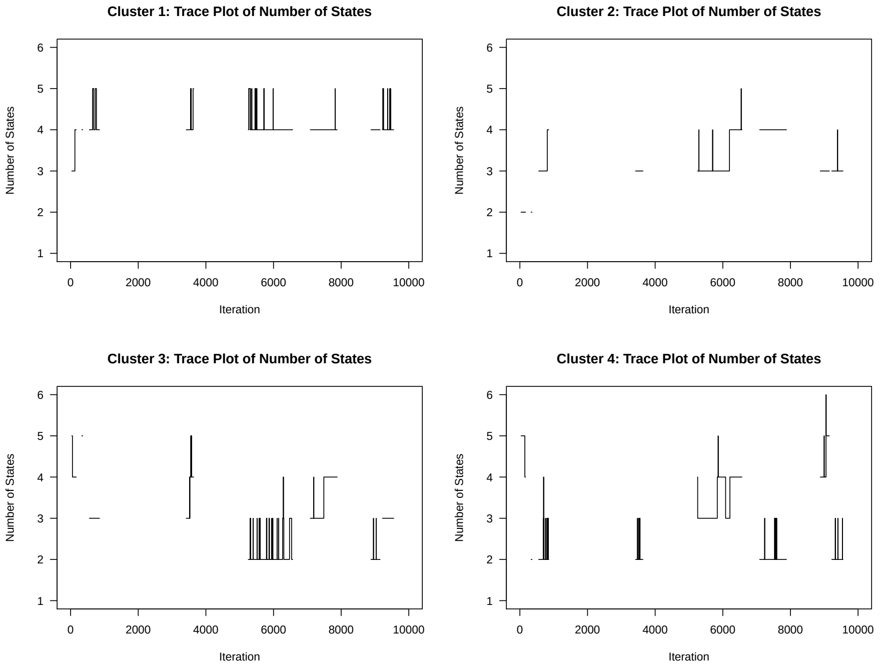

Fig. 9 Example 4: Trace plots of the number of states for Poisson case on four-cluster iterations.

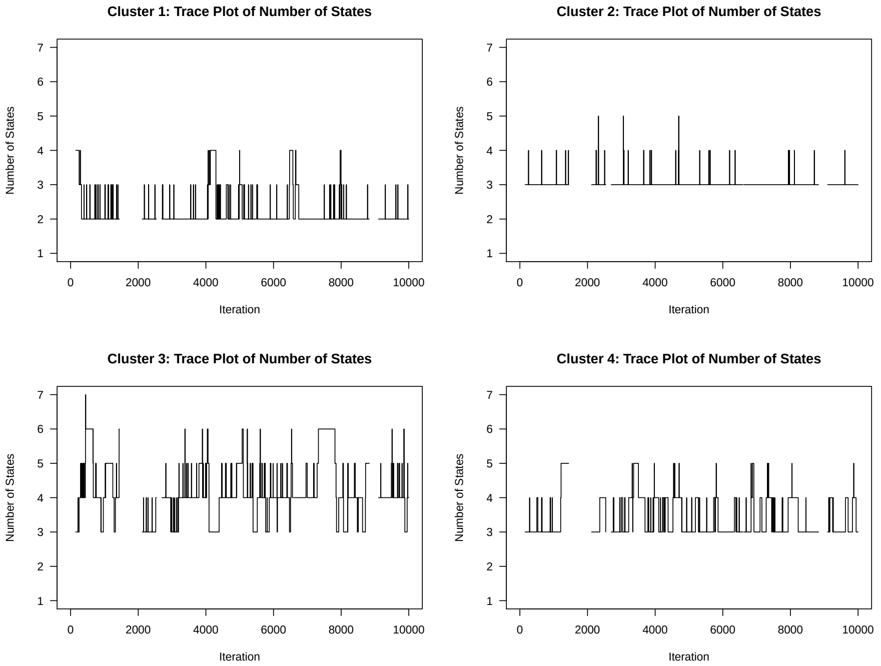

Supplement: Supplementary file 2 — Supplementary material 2 (pdf 1683 KB) [file 11222_2021_10032_MOESM2_ESM.pdf]
